# Occupational blood and body fluid exposures and human immunodeficiency virus post-exposure prophylaxis amongst intern doctors

**DOI:** 10.4102/HIVMED.v20i1.958

**Published:** 2019-05-22

**Authors:** Sunday J. Aigbodion, Feroza Motara, Abdullah E. Laher

**Affiliations:** 1 Department of Emergency Medicine, Far East Rand Hospital, Johannesburg, South Africa; 2 Department of Emergency Medicine, Faculty of Health Sciences, University of the Witwatersrand, Johannesburg, South Africa

**Keywords:** Occupational blood and body fluid exposure, Needle stick injury, Intern doctors, Post-exposure prophylaxis, Healthcare workers

## Abstract

**Background:**

Healthcare workers (HCWs) are constantly vulnerable to occupational blood and body fluid exposures (OBBFEs). Exposed HCWs experience emotional, physical and psychological trauma. Less experienced HCWs, such as intern doctors, are more prone to OBBFEs.

**Objectives:**

The aim of this study was to investigate the prevalence and practices pertaining to OBBFEs amongst a select group of intern doctors in the Gauteng province of South Africa.

**Methods:**

A quantitative cross-sectional descriptive study using a questionnaire based on a practical model was used. Intern doctors were recruited from four major hospitals in Gauteng.

**Results:**

A total of 175 intern doctors participated in the study. There was a total of 182 (mean = 1.04, standard deviation [s.d] 0.88) reported OBBFEs amongst 136 (77.7%) subjects. The exposures occurred predominantly whilst subjects were working in surgery (*n* = 50, 27.5%), obstetrics and gynaecology (*n* = 49, 26.9%) and internal medicine (*n* = 48, 26.4%) departments; were superficial wounds (*n* = 69, 37.9%); were acquired during vascular puncture or intravenous line insertion (*n* = 69, 37.9%); and occurred when subjects were working >12 h shifts (*n* = 101, 55.5%). Human immunodeficiency virus (HIV) post-exposure prophylaxis (PEP) was initiated in 141 (77.5%) out of the 182 exposures. Only 90 (63.8%) subjects completed the recommended 28-day course of PEP. Two (1.1%) subjects reported that they had acquired HIV infection as a consequence of the OBBFE.

**Conclusion:**

Occupational blood and body fluid exposures are common amongst intern doctors. It is recommended that regular training, health education and monitoring compliance should be incorporated during the induction of medical intern doctors in hospitals. The availability of PEP regimens with better tolerability will encourage compliance.

## Introduction

Medical doctors, especially intern doctors who are the most junior doctors employed at hospitals, face the threat of occupational blood and body fluid exposure (OBBFE) with the consequent risk of acquiring blood-borne infections (BBIs) by pathogens such as the human immunodeficiency virus (HIV), hepatitis B virus (HBV) and hepatitis C virus (HCV).^1^

Significant occupational exposure to blood and other infectious body fluids is defined as (1) percutaneous exposures resulting in a breach to the skin by a human bite or a contaminated needle, blade, lancet or other sharp objects; (2) mucocutaneous exposure which includes splashes to mucosal surfaces such as the nose, mouth or eyes; and (3) non-intact skin exposure which includes dermatitis, chapped skin, abrasions and open wounds. Potentially infectious body fluids include blood, tissue, semen, vaginal secretions, visibly bloody fluids as well as cerebrospinal, pleural, pericardial, synovial and amniotic fluids.^2^

Previous studies predominantly reported on OBBFEs or needle stick injuries (NSIs) amongst healthcare workers (HCW) in general.^3,4,5^ However, more recent studies have recognised the necessity of the frequent education of intern doctors concerning blood and body fluid exposures.^6^ Therefore, the rationale for this study was prompted by the lack of studies directed specifically at intern doctors. We hypothesised that intern doctors, because of their lack of experience, high workload and long working shifts,^7^ are more prone to OBBFEs. The aim of this study was to determine the prevalence, risk factors, adherence to post-exposure prophylaxis (PEP) guidelines and compliance with PEP amongst intern doctors.

## Methods

A quantitative cross-sectional descriptive study using a questionnaire based on a practice model was used. The study population comprised intern doctors employed at four different hospitals (Charlotte Maxeke Johannesburg Academic Hospital, Chris Hani Baragwanath Academic Hospital, Far East Rand Hospital and Thelle Mogoerane Hospital) in the Gauteng province of South Africa. The former two hospitals are tertiary academic hospitals based in central and southern Gauteng, respectively, whereas the latter two hospitals are secondary level regional hospitals based in the east of Gauteng. All four hospitals are affiliated with the University of the Witwatersrand.

Data collection was commenced soon after protocol approval and ethical clearance (University of the Witwatersrand, certificate no. M170496) were obtained. Data collection was conducted between 13 September and 19 December 2017. As the 2-year medical internship programme in South Africa generally commences on 01 January every year, it was assumed that all study participants had at least 8 months of working experience at the time of data collection. The first part of the questionnaires aimed to determine gender, age, experience, significant blood or body fluids exposures, reporting of these exposures and awareness of PEP protocols. The second part of the questionnaire was based on details of OBBFEs and actions taken after each exposure.

The primary researcher attended unit meetings at various clinical departments of the included hospitals where intern doctors were working. Participant information and informed consent sheets as well as questionnaires that were placed in an anonymous envelope were distributed to the intern doctors who were requested to voluntarily participate in the study. Completed questionnaires were placed back in the envelopes and collected immediately thereafter. Confidentiality and anonymity of the participants were maintained throughout the study.

Data were captured in an Excel spreadsheet (Microsoft^®^ Excel^®^ 2010) and imported into Stata version 14 (StataCorp^®^ 2015, College Station, TX) statistical software for analysis. Data were described and categorical variables were expressed as frequencies and percentages. The prevalence of blood and body fluid exposures was calculated. Clinical and socio-demographic characteristics of the participants were compared between those who were exposed and those who were not exposed to OBBFEs as defined in the questionnaire. Pearson’s chi-squared test and Fisher’s exact test were utilised to determine significant differences. The level of statistical significance was set at *p* < 0.05.

## Ethical consideration

Ethical approval for this study was obtained from the University of Witwatersrand Human Research Ethics Committee (certificate no. M170496).

## Results

A total of 212 questionnaires were administered, of which 175 were returned, giving a response rate of 82.5%. Out of the 175 subjects who completed the questionnaire, there was a total of 182 (mean = 1.04, standard deviation [s.d.] 0.88) reported OBBFEs amongst 136 (77.7%) subjects. Of these 136 subjects, 106 (77.9%) had one exposure, 21 (15.4%) had two exposures, 4 (2.9%) had three exposures, 3 (2.2%) had four exposures and 2 (1.5%) had five exposures. Therefore, a total of 30 (22.1%) subjects had reported more than one OBBFE.

Table 1 describes and compares gender, age group, work experience as well as the familiarity and user-friendliness with institutional OBBFE protocols and policies between subjects who had and those who had not experienced an OBBFE. Overall, there were marginally more female (*n* = 97, 55.4%) than male subjects, with almost all subjects being between 24 and 30 years of age (*n* = 170, 97.1%). The majority of subjects (*n* = 124, 70.9%) were working in their second year of medical internship. Only 40% of subjects (*n* = 70) reported that they were fully familiar with institutional OBBFE protocols/policies and 126 (72.0%) believed that these protocols/policies were user-friendly (*n* = 126, 72.0%). The majority of subjects were aware of where to report an OBBFE (*n* = 145, 82.8%) and were also aware of where to acquire the antiretroviral therapy starter pack after an exposure (*n* = 148, 84.6%). There were no statistically significant differences between those who had and those who had not experienced an OBBFE.

**TABLE 1:** Description and comparison of gender, age group, work experience as well as the familiarity and user-friendliness with institutional occupational blood and body fluid exposure protocols/policies between subjects who had and those who had not experienced an occupational blood and body fluid exposure.

Variables	Experienced OBBFE (*n* = 136)			Did not experience OBBFE (*n* = 39)		p*
	*n*	%		*n*	%	
**Gender**						
Male (*n* = 78, 44.6%)	57	41.9		21	53.8	0.186
Female (*n* = 97, 55.4%)	79	58.1		18	46.2	
**Age group (years)**						
24–30 (*n* = 170, 97.1%)	134	98.5		36	92.3	0.059
31–40 (*n* = 3, 1.7%)	1	0.7		2	5.1	
> 40 (*n* = 2, 1.1%)	1	0.7		1	2.6	
**Work experience**						
≤12 months (*n* = 51, 9.1%)	37	27.2		14	35.9	0.292
> 12 months (*n* = 124, 70.9%)	99	72.8		25	64.1	
**Familiarity with institutional OBBFE protocol/policy**						
Fully familiar (*n* = 70, 40.0%)	54	39.7		16	41.0	0.065
Partially familiar (*n* = 103, 58.9%)	82	60.3		21	53.8	
Don’t know that these exist (*n* = 2, 1.1%)	0	0.0		2	5.1	
**User-friendliness of institutional OBBFE protocol/policy**						
Yes (*n* = 126, 72.0%)	96	70.6		30	76.9	0.806
No (*n* = 27, 15.4%)	22	16.2		5	12.8	
I don’t know (*n* = 22, 12.6%)	18	13.1		4	10.3	

OBBFE, occupational blood and body fluid exposure.

* Statistical significance (*p* < 0.05).

The majority of exposures occurred whilst working in surgery (*n* = 50, 27.5%), obstetrics and gynaecology (*n* = 49, 26.9%) and internal medicine (*n* = 48, 26.4%) departments, with superficial wounds (no blood seen) being the most common wound type (*n* = 69, 37.9%). The majority of exposures were acquired during vascular puncture and/or intravenous line insertion (*n* = 69, 37.9%) and occurred when subjects were working > 12 h shifts (*n* = 101, 55.5%). More than three-quarter of exposures were reported within 24 h of the incident (*n* = 152, 83.5%). Only 149 (81.9%) subjects had a follow-up blood test done after the exposure (see Table 2).

**TABLE 2:** Description of the 182 occupational blood or body fluid exposures amongst study subjects.

Variables	*n*	%
**Department where exposure occurred**		
Surgery	50	27.5
Obstetrics and Gynaecology	49	26.9
Internal Medicine	48	26.4
Emergency Department	21	11.6
Paediatrics	8	4.4
Orthopaedics	3	1.6
Anaesthesia	3	1.6
Psychiatry	0	0.0
**Use of personal protective equipment (PPE) during exposure**		
Gloves	177	97.3
Goggles	30	16.5
Face mask	26	14.3
Plastic apron	20	10.9
**Procedure being performed or aetiology of exposure**		
Vascular puncture/intravenous line insertion	69	37.9
Wound suturing	43	23.7
Assisting in surgical procedures	21	11.6
Putting up a chest drain	11	6.0
Recapping used needles	11	6.0
Overfilled sharps container	11	6.0
Putting blood into specimen bottle	10	5.5
Amniotic fluid splash	6	3.3
**Nature of exposure**		
Superficial wound (blood not seen)	69	37.9
Deep wound (blood seen)	63	34.6
Mucocutaneous exposure	38	20.9
Non-intact skin exposure	12	6.6
**Action taken after exposure**		
Follow each step of local policy	87	47.8
Wash exposure area and/or replace gloves and continue	73	40.1
Ignore and continue	22	12.1
**Circumstances around exposure**		
Working shift > 12 h	101	55.5
Tiredness/fatigue	37	20.2
Working without supervision	19	10.4
Poor instruments	16	8.8
No assistant present	7	3.8
Poor lightings	2	1.1
**How soon after the incident was the exposure reported?**		
Within 24 h	152	83.5
24–48 h	10	5.5
> 72 h	12	6.6
48–72 h	6	3.3
Didn’t report the incident	2	1.1
**Was a follow-up blood test done?**		
Yes	149	81.9
No	33	18.1
**Infection acquired after the exposure**		
None	180	98.9
HIV	2	1.1
Hepatitis B or C	0	0.0

Two (1.1%) subjects reported that they had acquired HIV infection after the exposure. Both of these subjects reported HIV seroconversion on repeat testing within 4 months of the exposure. Both subjects were men between the ages of 24 and 30 years, were working in their second year of medical internship, sustained a deep needle stick injury to their finger, reported the incident within 24 h, initiated and completed the 28-day two-drug regimen of antiretroviral therapy, did not use a third antiretroviral agent and were wearing gloves. The first subject sustained his injury from a hollow bore needle whilst inserting an intravenous line, whilst the second subject sustained his injury from a suturing needle whilst inserting an intercostal drain. Unfortunately, the questionnaire did not enquire whether participants engaged in risky sexual behaviour around the time of the exposure.

**FIGURE 1:**
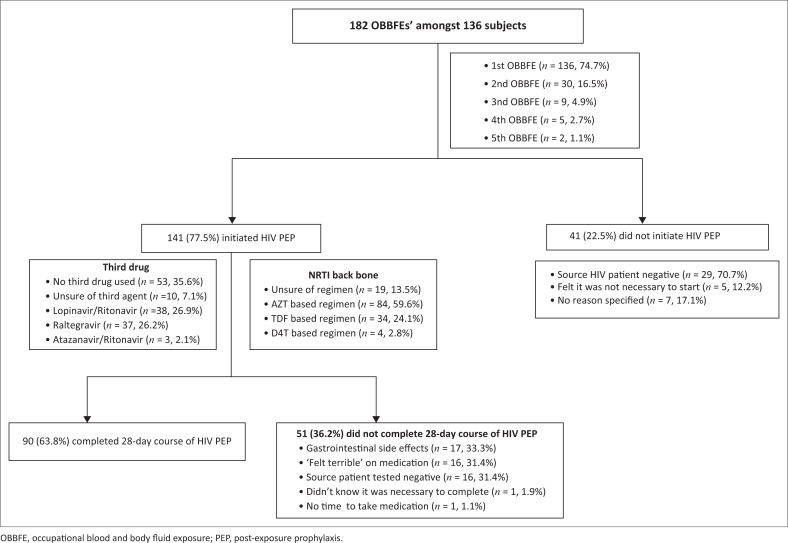
Initiation, compliance and regimen pertaining to HIV post-exposure prophylaxis amongst study subjects.

Initiation of antiretroviral therapy, drug regimens used and compliance with HIV PEP are described in Figure 1. Overall, HIV PEP was initiated in 141 (77.5%) out of the 182 exposures. However, the recommended 28-day course of therapy was only completed in 90 (63.8%) out of the 141 cases where PEP was initiated.

Amongst the 136 subjects who had experienced their first exposure, 107 (78.7%) had initiated and 68 (63.6%) had completed the 28-day course of PEP. Amongst the 30 subjects who had experienced their second exposure, 22 (73.3%) had initiated and 14 (63.6%) had completed PEP. Amongst the nine subjects who had experienced their third exposure, eight (88.9%) had initiated and five (62.5%) had completed PEP. Amongst the five subjects who had experienced their fourth exposure, three had initiated and two (66.7%) had completed PEP. Amongst the two subjects who had experienced their fifth exposure, one (50.0%) had initiated and completed PEP.

More than one-third (*n* = 51, 36.2%) of subjects who had initiated HIV PEP did not complete the 28-day course of therapy. The most common reasons for not completing the therapy included gastrointestinal side effects (*n* = 17, 33.3%), the subject ‘felt terrible’ on medication (*n* = 16, 31.4%) and the source patient tested negative for HIV (*n* = 16, 31.4%). The most commonly used PEP regimen was AZT based (*n* = 84, 59.6%) and at least 53 (35.6%) subjects did not use a third drug. Overall, 33 (18.1%) subjects were not aware of PEP regimens with less side effects.

## Discussion

Compared to other studies where the response rate ranged between 42% and 98%,^8,9,10,11,12,13^ the overall response rate in this study was 82.5%. Of the 182 OBBFEs, 98.9% were reported to the relevant authorities, with the majority (83.5%) being reported within 24 h of the exposure. A report rate of 77% was found in a study in Montenegro,^14^ compared with a lower report rate of 30% amongst medical trainees in East Toronto General Hospital.^8^ Kassa et al. in Botswana also found a low report rate of 37%.^15^ A much lower report rate of 28.9% was found in Ghana.^16^ The reason for the higher report rate amongst participants in this study could be attributed to greater awareness because of the high prevalence of HIV in South Africa.^17^

More than three-quarters (77.7%) of study participants had experienced at least one OBBFE. Internationally, the prevalence of NSIs has been reported as between 20.9% and 74%.^3,4,5^ In a large meta-analysis that included 16 105 HCWs from 44 studies across Iran, the overall prevalence of NSI was noted as 42.5% (95% CI 37% – 48%).^18^ Compared to our study that only included intern doctors, these studies included all HCWs (nurses, doctors, paramedics and laboratory staff). The higher prevalence in our study can be ascribed to the fact that intern doctors are relatively inexperienced, are frequently the first line of patient contact and are often left with the responsibility of performing basic procedures such as insertion of intravenous lines, wound suturing and taking of blood specimens. Also, in this study, most NSIs occurred whilst performing vascular puncture and/or intravenous line insertion or wound suturing (61.6% of cases). Comparatively, recapping used needles was found to be the reason for most NSIs in an Italian study,^19^ whereas in Toronto, Canada, Ben Ouyang et al. reported that most NSIs took place whilst performing wound suturing.^8^

There were no significant differences between those who had and those who had not experienced an OBBFE with regard to gender, age group, work experience, familiarity and user-friendliness with institutional OBBFE protocols/policies. However, bearing in mind that South Africa has a high burden of HIV, with approximately one-fifth of the adult population testing positive,^17^ it is concerning that more than half of the study participants were not fully familiar with institutional protocols/policies.

Globally, it is estimated that OBBFEs are responsible for approximately 66 000 HBV, 16 000 HCV and 200–5000 HIV infections amongst HCWs annually.^20^ The risk of HIV transmission has been estimated as 0.3% after percutaneous exposure and 0.1% after mucocutaneous exposure to HIV-infected blood.^21^ A meta-analysis that included 5810 patients from 22 studies reported a pooled infectivity estimate of 0.23% (95% CI: 0.00–0.46), with 15 of these studies reporting a transmission risk of 0%.^22^ Comparatively, the seroconversion rate was four- to fivefold higher in this study (*n* = 2, 1.1%). Both the subjects had completed the 28-day PEP regimen. However, a two-drug and not a three-drug regimen was prescribed in both cases. Although most PEP guidelines recommend a three-drug regimen, there is no evidence to suggest that a three or more drug regimen is superior to a two-drug regimen.^23^

More than half (55.5%) of the OBBFEs in this study occurred when subjects were working shifts of > 12 h, with 20.2% of subjects reporting tiredness and fatigue as a contributory factor. These findings are in line with the findings of a large systematic review that included 65 studies from 21 African countries. The authors concluded that a lack of training and working more than 40 h per week were risk factors for acquiring an OBBFE.^24^

A long-standing tradition in healthcare institutions is strict adherence and advocacy for the use of personal protective equipment (PPE) to minimise contact with blood and body fluids. Such measures include, but are not restricted to, the use of gloves, goggles and aprons. Most of the participants in this study used gloves (97.3%), which is considerably higher than the findings of other studies that reported figures of 89%^12^ and 52%.^25^

Poor adherence to standard PEP protocols has been associated with a high risk of seroconversion to HIV and other pathogens.^26,27^ One study reported that 45% of HCWs did not use PEP after an OBBFE, whilst 50% in the same study had to purchase the PEP themselves.^28^ This research study reported initiation of PEP amongst 77.5% of exposed respondents, which is suboptimal in a setting where HIV is endemic. Reasons for the suboptimal initiation of PEP include a lack of insight as well as a lack of strict enforcement and education regarding PEP protocols.^27,29^ Following education and implementation of institutional protocols, PEP uptake was observed to have a trajectory increase from 12% to 90% in a study by Peponis et al.^27^

This study revealed a PEP completion rate of 63.8% as compared to 79% recorded by Kassa et al.^15^ Established reasons for non-completion of PEP include fatigue, gastrointestinal side effects and drug rash, amongst others.^30^ Amongst the 77.5% of subjects who started PEP, 36.2% did not complete the course, predominantly because of poor tolerability of drug side effects. In light of this discussion, we strongly recommend that there should be psychological, social and emotional supports to exposed HCWs who are using PEP.

Of further concern is that 18.1% of subjects were unaware of alternate PEP regimens with better tolerability. The World Health Organization advocates the use of PEP regimens containing a third drug with improved tolerability and higher associated completion rates (e.g. dolutegravir, duranvir and raltegravir).^31^ Because of cost implications, these newer and more tolerable drug regimens have only recently been made available in some public sector hospitals in South Africa (pers. comm. with National Department of Health personnel). Even though these regimens are more expensive, their higher cost cannot be compared to the cost of reduced tolerability, which may lead to days off and the risk of acquiring HIV associated with not completing the entire 28-day course of PEP.^27^ Widespread availability of these newer PEP regimens in public hospitals would go a long way in ensuring PEP completion.

In summary, this study highlights the high prevalence of OBBFEs amongst intern doctors. We strongly advocate for better working hours for junior doctors, the widespread availability of triple drug PEP regimens with more tolerable side effect profiles and strict adherence to approved institutional guidelines. We also recommend the implementation of targeted educational programmes and the training of junior HCWs on local policies and guidelines relating to OBBFEs. Furthermore, healthcare institutions in conjunction with the National Department of Health should ensure that these strategies comply with locally and internationally acceptable standards and recommendations.

## Limitations

This is a regional study that was conducted in four hospitals in Gauteng province. Hence, the findings may not be representative of other regions in South Africa. Furthermore, other limitations that are associated with questionnaire-based studies also apply here, which include recall bias and selective non-disclosure.

## Conclusion

Occupational blood and body fluid exposures are common amongst intern doctors. It is recommended that regular training, health education and monitoring compliance should be incorporated during the induction of medical internship doctors in hospitals. The availability of PEP regimens with better tolerability will encourage compliance.
